# A numerical study of fish adaption behaviors in complex environments with a deep reinforcement learning and immersed boundary–lattice Boltzmann method

**DOI:** 10.1038/s41598-021-81124-8

**Published:** 2021-01-18

**Authors:** Yi Zhu, Fang-Bao Tian, John Young, James C. Liao, Joseph C. S. Lai

**Affiliations:** 1 School of Engineering and Information Technology, University of New South Wales, Canberra, ACT 2600 Australia; 2 Whitney Laboratory for Marine Bioscience, Department of Biology, University of Florida, Gainesville, FL 332611 USA

**Keywords:** Mechanical engineering, Fluid dynamics

## Abstract

Fish adaption behaviors in complex environments are of great importance in improving the performance of underwater vehicles. This work presents a numerical study of the adaption behaviors of self-propelled fish in complex environments by developing a numerical framework of deep learning and immersed boundary–lattice Boltzmann method (IB–LBM). In this framework, the fish swimming in a viscous incompressible flow is simulated with an IB–LBM which is validated by conducting two benchmark problems including a uniform flow over a stationary cylinder and a self-propelled anguilliform swimming in a quiescent flow. Furthermore, a deep recurrent Q-network (DRQN) is incorporated with the IB–LBM to train the fish model to adapt its motion to optimally achieve a specific task, such as prey capture, rheotaxis and Kármán gaiting. Compared to existing learning models for fish, this work incorporates the fish position, velocity and acceleration into the state space in the DRQN; and it considers the amplitude and frequency action spaces as well as the historical effects. This framework makes use of the high computational efficiency of the IB–LBM which is of crucial importance for the effective coupling with learning algorithms. Applications of the proposed numerical framework in point-to-point swimming in quiescent flow and position holding both in a uniform stream and a Kármán vortex street demonstrate the strategies used to adapt to different situations.

## Introduction

It has long been observed that fish can adapt to different environments and achieve their goals optimally. These adaption behaviors are essential for survival since they allow a fish to obtain and save energy as well as avoid risks. A typical example of adaption behavior is prey capture, in which the fish is trying to reach a target with given time (generalized as point-to-point swimming). Another important behavior that has been observed in many fishes is rheotaxis^1,2^, which is a tendency of the fish to directly face into an oncoming current to capture food carried by the flow. Furthermore, a unique energy-saving behavior termed Kármán gaiting is observed in rainbow trout and other fishes when swimming behind a bluff body in the flow, which is characterized by large-amplitude lateral motion of the body occurring at a low frequency^3–5^. In addition, fish may exploit the vortices shedding from its leading one or its fins to improve its swimming performance^6–8^, of which the propulsion mechanism can be further revealed by separating the drag and thrust^9^. In nature, fish are able to achieve the above mentioned behaviors in a very quick and efficient way, with which current man-made vehicles cannot compete. Therefore, it is important to achieve them in numerical simulation, with which researchers are able to understand the design concepts of fish, and to put these concepts into man-made vehicles.

The mechanisms underlying these adaption behaviors are complex and have not been fully understood. The cooperation between the sensory system, the neural system and the muscles, which forms a precise and robust feedback control system, is of primary importance. The sensory system is responsible for continuously collecting information about the environment which is input into the neural system so that the fish can update its knowledge of its surroundings in real time. Based on this information, the fish may change its swimming kinematics via the muscles to achieve its goals. During over 400 millions years of evolution, a variety of sensory systems have emerged in different fishes based on proprioceptive, visual, tactile, olfactory, electric and hydromechanical signals^10–12^. Among them, visual signals from the eyes and hydrodynamical signals from the lateral line system are the most commonly used, allowing fish to perform a variety of adaption behaviors^13,14^.

Significant effort has been directed towards reproducing the adaption behaviors. Feedback control design has been adopted in robot fish^15,16^ based on the correlation between the pressure on the fish body and the position with respect to an object or a point in the flow field. The effect of a specific action in the control problem is typically predicted by using a simplified flow dynamics model (e.g. an inviscid potential flow model). However, the development of a robust and accurate controller is still a challenging problem, due to incomplete flow models and nonlinear historical influence of past actions. Given the difficulty of acquiring a reliable policy to reproduce adaption behaviors, swimmers in numerical simulations are often forced to swim in preferable configurations, thus making them not entirely free swimming^17–20^.

In order to address the challenge in developing robust and accurate controllers, a novel control method based on reinforcement learning (RL) has been proposed to study bio-inspired swimming and flying problems including the individual^21^ or the collective motion of fish^22,23^ and dipole swimmers^24^, autonomous thermal soaring of UAVs^25,26^ and birds^27,28^, lift generation of UAVs^29–32^, and the navigation of microswimmers^18,33^. The method has two remarkable advantages. The first advantage is that the swimmer does not need to possess any prior knowledge of the environment. Instead, it only needs to sample the information about the environment through trial and error and so there is no need to simplify the flow dynamics. The other advantage is that the influence of the historical states can be easily taken into consideration. Therefore, the correlation between action and its effect can be accurately captured even when there is a delay between them and there are measurable historical impacts from historical actions.

A challenge of the method is that in order to obtain a robust control policy, the learning agent must repetitively explore a large number of different possible actions in many environment states. Thus, an efficient way to obtain the environmental flow information is of crucial importance for the agent to learn in a reasonable time, which is a great challenge for numerical simulations^34^. Here the environmental flow information is updated by using an immersed boundary–lattice Boltzmann method (IB–LBM) which makes excellent use of the advantages of the lattice Boltzmann method (LBM) and the immersed boundary method (IBM)^35–41^. Compared to traditional numerical methods based on the Navier–Stokes equations, the IB–LBM is more efficient^42,43^ and is a promising alternative in combining with reinforcement learning methods. The IBM is a methodology for dealing with boundary conditions at interfaces based on meshes that do not conform to the shapes of the immersed boundaries. In the IBM, the mesh generation is very easy even for complicated geometries. The mesh movement and mesh regeneration are not necessary for flows involving moving boundaries and fluid–structure interaction (FSI) problems. Therefore, it is very convenient to handle cases involving topological change of the computational domain, complicated geometries and large movement of boundaries^36,40,44–48^.

In this work, a deep recurrent Q-network (DRQN) is coupled with an adaptive-mesh IB–LBM FSI solver for the simulation of the FSI system mimicking fish adaption behaviors including prey capture in still water, rheotaxis in a uniform flow and Kármán gaiting in a Kármán vortex street. It should be noted that we recognize each component in the computational framework is not new. However, the combination of DRQN and adaptive IB–LBM FSI solver provides a very efficient tool to study the fish behaviours in complex environments. In addition, a new mathematical model of fish-like swimming is developed with amplitudes and frequencies altered smoothly every half period to implement highly maneuverable motions for the swimmer swimming in highly complex and dynamic flow environments. The feasibility and efficiency of the combined DRQN and IB–LBM method will be demonstrated by applying it in fish behaviors in three typical cases.

The rest of this paper is organized as follows. Numerical models, IB–LBM and DRQN are introduced in “Numerical model and methodology” section. The flow solver is validated in “Validation of the fluid solver” section. Adaption behaviors are discussed in “Applications of the coupled DRQN and IB–LBM” section and the conclusions are provided in “Conclusions” section.

## Numerical model and methodology

### The shape and motion of a trout model

**Figure 1:**
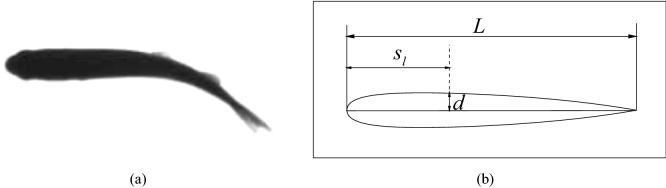
A schematic of the fish model: (**a**) ventral view of a trout^49^; and (**b**) the constructed shape of a 2D model.

The shape of the 2D swimmer model here is reconstructed from the cross-section of a trout^49^ as shown in Fig. 1. The half thickness of the body is mathematically approximated by

1 $$\begin{aligned} \frac{d}{L}=0.2610\sqrt{\frac{s_l}{L}}-0.3112\left( \frac{s_l}{L}\right) +0.1371\left( \frac{s_l}{L}\right) ^2-0.0791\left( \frac{s_l}{L}\right) ^3-0.0078\left( \frac{s_l}{L}\right) ^4, \end{aligned}$$

where *L* is the body length, and $s_l$ is the arc length along the mid-line of the body.

**Figure 2:**
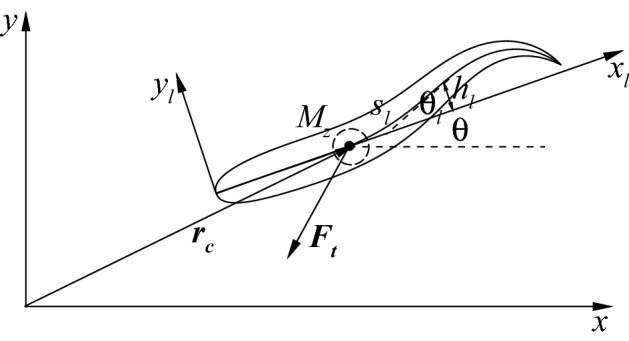
A schematic illustration of the motion of the swimmer.

The motion of the body includes three parts as shown in Fig. 2: the undulation motion of the body ($h_l$ in $x_l$–$y_l$ system), the translation of the mass center ($\varvec{r_c}$), and the body rotation around the mass center ($\theta$). The undulatory motion can be taken as the superposition of different waves propagating from head to tail. In order to implement the DRQN in an easy way as explained later, every wave lasts only half cycle. In the *n*-th half cycle, the mid-line lateral displacement is determined by

2 $$\begin{aligned}&h_l(s_l,t)=\int ^{s_l}_0 sin(\theta _l)ds, \end{aligned}$$

3 $$\begin{aligned}&\theta _l(s_l,t)=\theta _{lmax}\left( \frac{s_l}{L}\right) ^2h\left[ \frac{\lambda _n}{T_n}(t-t_{0n})-\frac{s_l}{L}\right] , \end{aligned}$$

where $\theta _l$ is the deflection angle of the mid-line with respect to axis $x_l$ as shown in Fig. 2, $\theta _{lmax}$ is the maximum deflection angle at the trailing edge, $\lambda _n$ is the wavelength, $T_n$ is the period, *t* is the time, $t_{0n}=0$ for $n=1$ and $\sum _1^{n-1}T_n$ for $n>1$, and *h* is the waveform function described by

4 $$\begin{aligned} h(\zeta )=c_{0}+c_{1}\zeta+c_{2}\zeta ^{2}+c_{3}\zeta ^{3}+c_{4}\zeta ^{4}+c_{5}\zeta ^{5}, \end{aligned}$$

where $c_{0-5}$ can be determined by $h(0)=h(\lambda _n/2)=1$, $h'(0)=h'(\lambda _n/2)=0$, $h''(0)=-(2\pi /\lambda _{n-1})^2$, and $h''(\lambda _n/2)=-(2\pi /\lambda _n)^2$. This undulatory motion is constructed based on extensive videos of rainbow trout free swimming, rheotaxis and Kármán gaiting^49–51^. It allows the swimmer to change its periods, amplitudes and wavelengths smoothly and arbitrarily every half period. Therefore, the swimmer model is able to choose appropriate combinations of different kinematics to achieve different maneuvering movements such as accelerating, decelerating and yawing, which enables the fish to handle complex and fast-changing environments.

The translational and rotational motions of the swimmer are determined by FSI in the global coordinate system (*x*, *y*) according to:

5 $$\begin{aligned}&m\frac{d^2\varvec{r_c}}{dt^2}=\varvec{F_t}, \end{aligned}$$

6 $$\begin{aligned}&\frac{d}{dt}\left( I_z\frac{d\theta }{dt}\right) =M_z, \end{aligned}$$

where *m* is the mass of the fish, $\varvec{{F_t}}$ is the total hydrodynamic force on fish body, $I_z$ is the inertia moment of the center of the mass, and $M_z$ is the total hydrodynamic torque on the center of the mass.

### IB–LBM for the fluid–structure–interaction system

An IB–LBM is adopted to solve the FSI system^52–54^. In this method, the fluid dynamics is obtained by solving the multiple-relaxation-time lattice Boltzmann equation,

7 $$\begin{aligned} f_i(\varvec{x}+\varvec{c}_i\Delta t,t+\Delta t)-f_i(\varvec{x},t)={\Omega }_i(\varvec{x},t)+\Delta tG_i(\varvec{x},t), \quad \quad i=0,\ldots ,8 \end{aligned}$$

where *f* is the distribution function, $\varvec{x}=(x,y)$ is the space coordinate, $\varvec{c_i}$ is the discrete lattice velocity, $\Delta t$ is time step, and ${\Omega }_i$ and $G_i$ are respectively the collision operator and the source term. Here ${\Omega }_i$ and $G_i$ are obtained by

8 $$\begin{aligned}&{\Omega }_i=-(M^{-1}SM)_{ij}(f_j-{f^{eq}}_j), \end{aligned}$$

9 $$\begin{aligned}&G_i=[M^{-1}(I-S/2)M]_{ij}F_j, \end{aligned}$$

where *M* is a $9\times 9$ transformation matrix, *S* is the relaxation matrix, *I* is the identity matrix, and $f^{eq}_i$ and $F_i$ are respectively the equilibrium distribution function and the effect of the fluid body force. $f^{eq}_i$ and $F_i$ are determined by

10 $$\begin{aligned}&{f^{eq}}_i={w}_i\rho \left[ 1+\frac{\varvec{u}\cdot \varvec{c}_i}{c^2_s}+\frac{(\varvec{u}\cdot \varvec{c}_i)^2}{2 c^4_s}-\frac{\varvec{u}\cdot \varvec{u}}{2 c^2_s}\right] , \end{aligned}$$

11 $$\begin{aligned}&F_i={w}_i\left( \frac{\varvec{c}_i-\varvec{u}}{c^2_s}+\frac{\varvec{c}_i\cdot \varvec{u}}{c^4_s}\varvec{c}_i \right) \cdot \varvec{g}, \end{aligned}$$

where $w_i$ is a weighting coefficient, $\rho$ is the density of the fluid, $\varvec{u}$ is the velocity of the fluid, $c_s=\Delta x/(\sqrt{3}\Delta t)$ is the lattice speed of sound, $\Delta x$ is the lattice spacing, and $\varvec{g}$ is the body force. In this work, D2Q9 is used. The *M* and *S* matrices of this model can be found in Lallemand and Luo^52^ and Krüger et al.^55^. $\varvec{c}_0$-$\varvec{c}_8$ are $(0,0),(\pm 1,0),(0,\pm 1),(\pm 1, \pm 1)$. $w_0=4/9$, $w_1$-$w_4=1/9$, and $w_5$-$w_8=1/36$.

Once the distribution function *f* is obtained, the macro fluid density $\rho$, velocity $\varvec{u}$, pressure *p*, viscous stress tensor $\sigma _{\alpha \beta }$ and fluid force density on the boundary $\varvec{F_{f}}$ in the new time step are calculated with

12 $$\begin{aligned}&\rho =\sum f_i,\quad p=\rho c^2_s, \quad \varvec{u}=\frac{1}{\rho }\left( \sum {f_i\varvec{c}_i}+\frac{\Delta t \varvec{g}}{2}\right) , \end{aligned}$$

13 $$\begin{aligned}&\sigma _{\alpha \beta }=-\sum {[M^{-1}(I-S/2)M]_{ij}(f_j-{f^{eq}}_j+F_j) c_{i\alpha }c_{i\beta }}, \end{aligned}$$

14 $$\begin{aligned}&F_{f\alpha }=(\sigma _{\alpha \beta }-p\delta _{\alpha \beta })n_{B\beta }, \end{aligned}$$

where $n_{B\beta }$ is the outer normal vector of the boundary $S_B$, $\delta _{\alpha \beta }$ is the Kronecker delta, and $\alpha$ and $\beta$ are dummy indices. The forces and moment of the fluid exerting on the swimmer model can be calculated with

15 $$\begin{aligned}&\varvec{F_t}=\int \limits _{S_B}\varvec{F_f}ds_0, \quad M_z=\int \limits _{S_B}\varvec{F_f}\times (\varvec{X}-\varvec{r_c})\cdot \varvec{e_z}ds_0, \end{aligned}$$

16 $$\begin{aligned}&F_D=\int \limits _{S_B}\varvec{F_f}\cdot \varvec{e_x}ds_0, \quad F_L=\int \limits _{S_B}\varvec{F_f}\cdot \varvec{e_y}ds_0, \end{aligned}$$

where $F_{D}$ is the drag, $F_{L}$ is the lift, $\varvec{X}$ is the Lagrangian coordinate on the fish surface, $s_0$ is the arc length along the surface of the swimmer, and $\varvec{e_x},\varvec{e_y}$ and $\varvec{e_z}$ are the unit vectors along *x*-axis, *y*-axis and yaw axis, respectively.

In addition, the IBM is utilized to handle the boundary condition at the fluid–structure interface according to

17 $$\begin{aligned}&\varvec{F_{IB}}(\varvec{X})=\eta \left[ \varvec{u_B}(\varvec{X})-\int \limits _{V_f}\varvec{u}(\varvec{x})\delta (\varvec{X}-\varvec{x})d\varvec{x}\right] , \end{aligned}$$

18 $$\begin{aligned}&\varvec{g_{IB}}(\varvec{x})=\int \limits _{S_B}\varvec{F_{IB}}(\varvec{X})\delta (\varvec{X}-\varvec{x})ds_0, \end{aligned}$$

where $\varvec{F_{IB}}$ is the Lagrangian force on the immersed boundary, $\varvec{g_{IB}}$ is the fluid body force due to the boundary, $S_B$ is the boundary surface of the rigid body, $\eta$ is the feedback coefficient, $\varvec{u_B}$ is the prescribed moving speed of the boundary surface, and $V_f$ is the fluid domain. The value of $\eta$ is determined by the geometry of the body, which can be found in Refs.^53,54^. $\delta$ is approximated with a kernel function,

19 $$\begin{aligned} \Delta (x,y)= & {} \frac{1}{\Delta x^2}\phi \left( \frac{x}{\Delta x}\right) \phi \left( \frac{y}{\Delta y}\right) , \end{aligned}$$

20 $$\begin{aligned} \varvec{\phi }(x)= & {} \left\{ \begin{array}{ll} \frac{1}{4}[1+cos(\frac{\pi x}{2})], \quad 0\le x\le 2, \\ 0, \qquad \qquad \qquad \quad 2\le x. \end{array} \right. \end{aligned}$$

Furthermore, a multi-block geometry-adaptive Cartesian grid is coupled with the IB–LBM to improve the computational efficiency. A detailed description of this grid structure and method can be found in Refs.^53,54^.

The fluid–structure system is coupled by an explicit FSI coupling according to,

21 $$\begin{aligned}&\frac{\varvec{r_c}^{i+1}-\varvec{r_c}^i}{\Delta t}=\frac{\varvec{u_c}^{i+1}+\varvec{u_c}^i}{2}, \end{aligned}$$

22 $$\begin{aligned}&m\frac{\varvec{u_c}^{i+1}-\varvec{u_c}^i}{\Delta t}=\varvec{F_t}^i, \end{aligned}$$

23 $$\begin{aligned}&\frac{\theta ^{i+1}-\theta ^i}{\Delta t}=\frac{\omega ^{i+1}+\omega ^i}{2}, \end{aligned}$$

24 $$\begin{aligned}&\frac{I_z^{i+1}+I_z^i}{2}\frac{\omega ^{i+1}-\omega ^i}{\Delta t}+\omega ^{i+1}\frac{I_z^{i+1}-I_z^i}{\Delta t}=M_z^i, \end{aligned}$$

where $\varvec{u_c}$ is the velocity of the mass center, and $\omega$ is the angular velocity. Since no iteration is required at each time step, this method is much more efficient than strong coupling methods^39,56^.

Yoshino et al.^43^ compared the computational efficiency between the LBM and the finite difference method (FDM) in modeling lid-driven cavity flows, and found that the CPU time of each step for the LBM is about 1/3 of that for the FDM, indicating the LBM is more efficient than the FDM in modeling fluid dynamics, which is of crucial importance for the coupling with the reinforcement learning method as each learning application normally requires thousands of simulation cases. The FSI process implemented by the IB–LBM is shown in Algorithm 1.

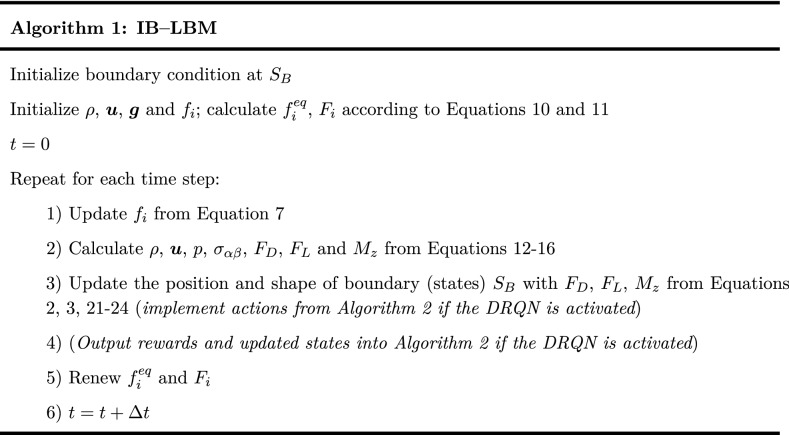



### Deep reinforcement learning

Deep reinforcement learning combines reinforcement learning with an artificial neural network to approach human-level control in complex real-world problems^57^. One of the most successful methods in reinforcement learning is one-step Q-learning. In this study, the DRQN^58^ is used where a one-step Q-learning is coupled with a three-layer long-short-term-memory recurrent neural network (LSTM-RNN).

Q-learning describes a general process of an agent learning how to achieve a goal during prolonged and continued interaction with its environment by trial and error^18^. During this process, the agent must be able to sense a defined set of parameters representing the state of the environment (denoted by *s*) and take actions (denoted by *a*) to affect it. Each action is assessed with a scalar number called the reward (denoted by *r*) whose value indicates whether the agent moves towards or away from the goal by taking the action. In order to achieve the goal, the agent must seek actions that maximize its expected cumulative rewards in the long run (also known as the action-value function) which is defined as,

25 $$\begin{aligned} Q(s_n,a_n)=E[r_{n+1}+\gamma r_{n+2}+\gamma ^2 r_{n+3}+\gamma ^3 r_{n+4}+\cdots \quad | \quad s_n,a_n], \end{aligned}$$

where $s_n$ and $a_n$ are respectively the *n*-th state and action, $r_{n+1}$ is the reward of *n*-th action, $r_{n+2}$, $r_{n+3}$ and $r_{n+4}$ are the subsequent rewards, and $\gamma$ is the discount rate ranging from 0 to 1. If $\gamma =0$, the agent is termed “myopic” because it only maximizes the immediate rewards. Larger $\gamma$ means that the agent is more “far-sighted”. For all cases in this paper, $\gamma$ is chosen to be 0.99 as in Ref.^57^. The principle in Q-learning is that the agent explores different actions in different states and evaluates the actions with *Q*(*s*, *a*), so that when the state reoccurs, the agent will choose the optimal action to achieve its goal.

Q-learning suffers from the classical “curse of dimensionality” problem, where the data and computational resource required grow exponentially with the dimensionality of the state and action spaces. Deep reinforcement learning has partly resolved this problem by approximating the action-value function with a neural network *Q*, which can generalize past experience to new situations^57^. In this work, an LSTM-RNN composed of three layers of 64 LSTM cells and a linear output layer is adopted. In order to find the optimal action-value function, the neural network is iteratively updated by minimizing the temporal difference error

26 $$\begin{aligned} TD_{err}=r_{n+1}+\gamma Q^*(s_{n+1},a^*_{n+1})-Q(s_n,a_n), \end{aligned}$$

where $Q^*(s,a)$ is the optimal (maximized) action-value function, i.e. $Q^*(s_{n+1},a^*_{n+1})=\max \limits _{a}Q(s_{n+1},a)$ for all actions in state $s_{n+1}$, and $a^*$ is the optimal action maximizing *Q*. This can be achieved by updating the network weights via gradient descent methods,

27 $$\begin{aligned} ws_i=ws_i-\alpha \frac{\partial {(TD_{err})}^2}{\partial ws_i}, \end{aligned}$$

where $ws_i$ is weight of the network, $\alpha$ is the learning rate. For efficient updating, the gradient descent is performed with the Adam optimization algorithm^59^.

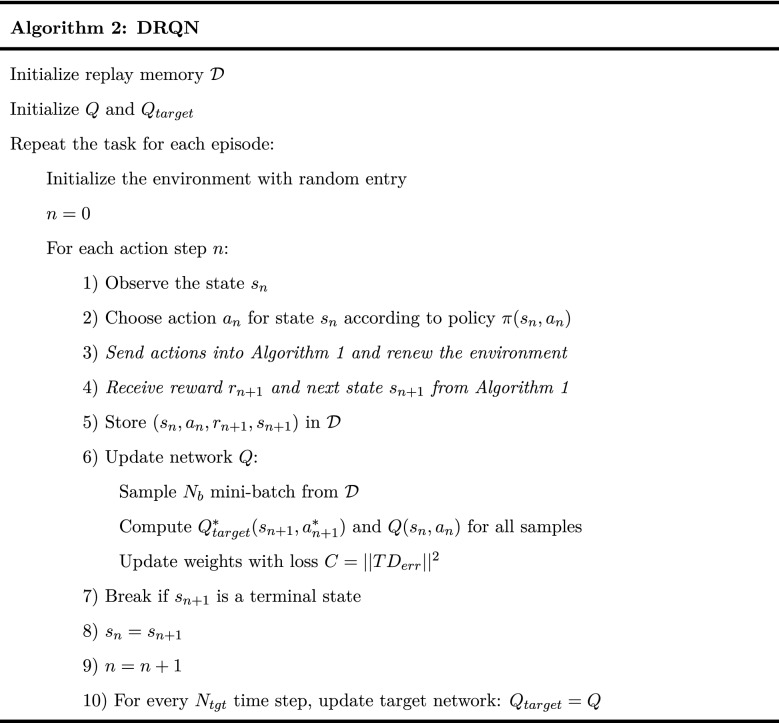



The state, action, reward, and next state quadruplet $(s_n,a_n,r_{n+1},s_{n+1})$ generated with agent-environment interaction are required to update the neural network. A replay memory $\mathcal {D}$ and a target neural network $Q_{target}$ are introduced in the iteration process^57^. The replay memory is used to store large numbers of quadruplets $(s_n,a_n,r_{n+1},s_{n+1})$ which are sampled randomly in a mini-batch $[\ldots ,(s_n^i,a_n^i,r_{n+1}^i,s_{n+1}^i),\ldots ]$ to update *Q*. This technique breaks the correlation between the samples to avoid local optimization^57^. The sizes of the replay memory ($N_{\mathcal {D}}$) and the mini-batch ($N_b$) are respectively set as $N_{\mathcal {D}}=5000$ and $N_b=100$. The target neural network is used to generate the optimal action value $Q^*(s_{n+1},a^*_{n+1})$ in Eq. (27). It is updated with *Q* for every $N_{tgt}$ action steps to avoid the instability caused by frequent update of the optimal action-value function^57^. $N_{tgt}$ is set to be 100. The learning parameters (i.e. $N_{\mathcal {D}}, N_b$ and $N_{tgt}$) have been tested to ensure the stability of the learning process.

The detailed interaction procedure is summarized in Algorithm 2 where the agent-environment interaction is broken into $N_e$ episodes. Each episode is divided into a sequence of discrete action steps $n=0,1,2,3,\cdots$. At step *n* of each action, the agent detects a state $s_n$, and selects an action $a_n$ based on a policy $\pi (s,a)$ which describes the probability of selecting each possible action in each state. At action step $n+1$, in response to the action $a_n$, the agent receives a reward $r_{n+1}$, and finds itself in a new state $s_{n+1}$. The $\epsilon$-greedy policy^60^ is used to select actions, with which the agent chooses the optimal action (also known as exploiting) with probability $1-\epsilon$ and other actions (also known as exploring) with probability $\epsilon$. $\epsilon$ gradually decays from 1 to 0.05 so that the agent explores more at the initial stage of the simulation but exploits more in the long term afterwards.

It should be noted that compared to existing models of learning for fish^21–23^, this work incorporates the fish position, velocity and acceleration into the state space in the DRQN; and it considers the amplitude and frequency action spaces as well as the historical effects.

## Validation of the fluid solver

The current flow solver has been validated in previous publications^53,54^. Here we further provide application-specific validations by focusing on the frequency of vortex shedding from a cylinder in a uniform flow and the swimming speed of an anguilliform swimmer in a quiescent flow. The cases are conducted with 20 computational cores on a workstation with Intel Xeon CPU E5-2650 and OpenMP.

### A uniform flow over a stationary cylinder

A uniform flow over a stationary cylinder is conducted to determine the frequency *f* of the Kármán vortex street by varying the Reynolds number $Re=\rho UD/\mu$ from 60 to 360, where $\rho$ is the density of the fluid, *U* is the incoming fluid velocity, *D* is the diameter of the cylinder, and $\mu$ is the dynamic viscosity of the fluid. The computational domain of $50D\times 50D$ is divided into 7 blocks with about $52.0\times 10^3$ points. The minimum nondimensional grid spacing is $\Delta x/D=\Delta y/D=0.01$ near the inner boundaries and the nondimensional time step size is $\Delta t U/D=0.01$. Validation has been performed to ensure the numerical results are independent of mesh size, domain size and time step size.

**Figure 3:**
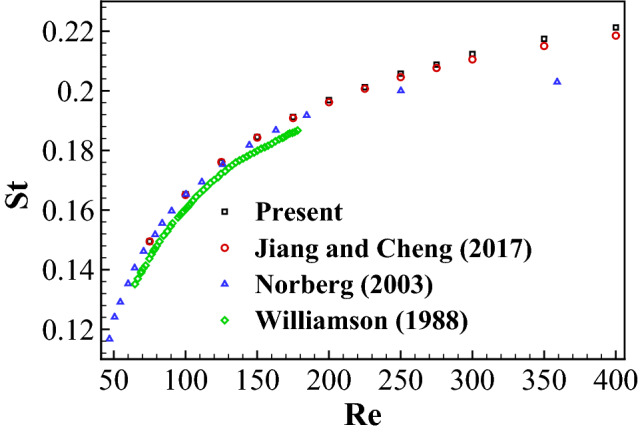
Strouhal number as a function of the Reynolds number for a uniform flow over a stationary cylinder.

The Strouhal number $St=fD/U$ of the vortex street computed by the present method and observed in Refs.^61–63^ is shown in Fig. 3. The mean drag coefficient $\bar{C_D}$ and the peak-to-peak lift coefficient $\Delta C_L$ at $Re=100$ are compared with other studies in Table 1. The simulation requires about 1.44s of CPU time per nondimensional time unit $tU/D=1.0$. Here the drag and lift coefficients are respectively defined by $C_D=F_D/(0.5\rho U^2D)$ and $C_L=F_L/(0.5\rho U^2D)$. Figure 3 and Table 1 show that *St*, $\bar{C_D}$ and $\Delta C_L$ predicted by our solver agree well with those in previous publications.

**Table 1:** Comparison of mean drag coefficient and peak-to-peak lift coefficient for a uniform flow over a stationary cylinder at $Re=100$.

	$\bar{C}_D$	$\Delta C_L$
Present	1.373	0.679
Shu et al.^64^	1.383	0.700
Tseng and Ferziger^65^	1.420	0.580
Lai and Peskin^66^	1.447	0.660
Liu et al.^67^	1.350	0.678

### Self-propelled anguilliform swimmer swimming in a quiescent flow

**Figure 4:**
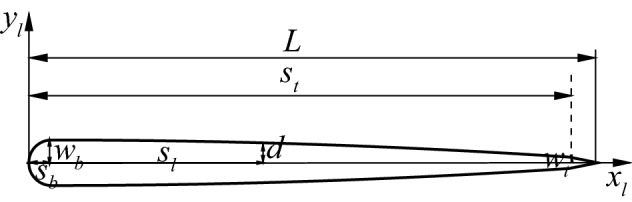
Geometry of a self-propelled anguilliform swimmer.

Here an anguilliform swimmer swimming in a quiescent flow is conducted to validate the capability of the current fluid solver for modelling a self-propelled swimmer. The half thickness of the swimmer is described as^68^

28 $$\begin{aligned} d = \left\{ \begin{array}{ll} \sqrt{2w_bs_l-s_l^2}, \quad 0\le s_l< s_b, \\ w_b-(w_b-w_t)(\frac{s_l-s_t}{s_t-s_b})^2, \quad s_b\le s_l<s_t, \\ w_t\frac{L-s_l}{L-s_t}, \quad s_t\le s_l \le L, \end{array} \right. \end{aligned}$$

where $w_b=s_b=0.04L$, $s_t=0.95L$ and $w_t=0.01L$ as shown in Fig. 4. To propel the swimmer, a travelling wave propagating from head to tail is generated,

29 $$\begin{aligned} y_l(s_l,t)=A_{max}\frac{s_l/L+0.03125}{1.03125}sin[2\pi (t/T-s_l/L)], \end{aligned}$$

where $A_{max}$ is the maximum waving amplitude at the tail tip, and *T* is the waving period. Body length of the fish *L*, fluid density $\rho$, and waving period *T* are chosen as the characteristic values. To compare with the result in Ref.^68^, the parameters are selected as: $A_{max}=0.125L$, and $Re=\rho L^2/T\mu =7142$. The translation and rotation are determined by Eqs. (5) and (6). The computational domain of $50L\times 50L$ is divided into 7 blocks with about $45.2\times 10^3$ initial points. The minimum nondimensional grid spacing is $\Delta x/L=\Delta y/L=0.01$ near the inner boundaries and the nondimensional time step size is $\Delta t /T=0.01$. The simulation requires about 2.41s of CPU time per nondimensional time unit $t/T=1.0$.

**Figure 5:**
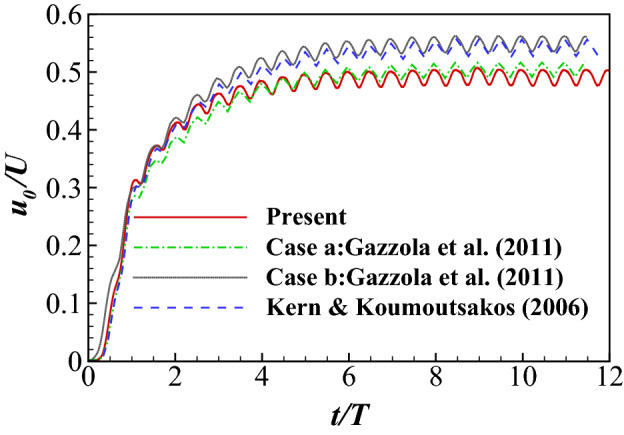
Time history of the forward swimming velocity of an anguilliform swimmer. Case a and Case b of Gazzola et al.^69^ are without and with divergence-free correction of body motion, respectively.

The forward velocity $u_0/U$ ($U=L/T$ is one body per waving period) predicted by the current solver is shown in Fig. 5 and compared with the results reported by Kern and Koumoutsakos^68^. It is noted that the balanced swimming velocity of the present study is smaller than that of Kern and Koumoutsakos^68^ and Case b (with divergence-free correction of body motion) of Gazzola et al.^69^, but agrees well with Case a (without divergence-free correction of body motion) of Gazzola et al.^69^. As the divergence of body motion does not affect the learning process considered in this work, and thus is not corrected in order to save computational costs.

## Applications of the coupled DRQN and IB–LBM

Three applications of the coupled DRQN and IB–LBM are conducted to demonstrate the effectiveness of this approach for the investigation of fish behaviors in different flow environments: point-to-point swimming in a quiescent flow mimicking prey capture behavior, position holding swimming in a uniform flow mimicking rheotaxis behavior and position holding in a Kármán vortex street behind a half-cylinder mimicking the Kármán gaiting behavior. The simulations are conducted with 20 computational cores on a workstation with Intel Xeon CPU E5-2650 with OpenMP.

### Point-to-point swimming

**Figure 6:**
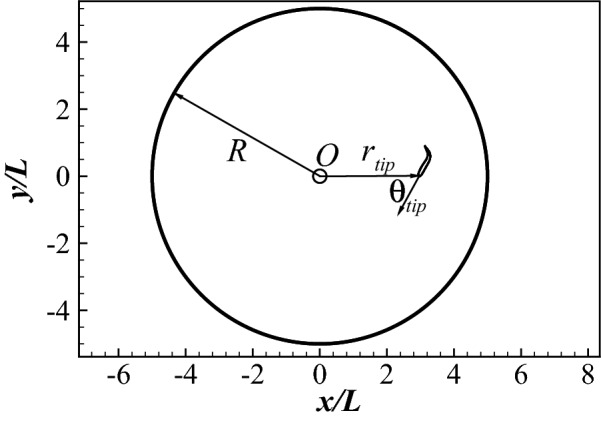
The confined domain of the point-to-point swimming.

Here we apply the coupled approach to the point-to-point swimming of a sub-carangiform swimmer in a quiescent flow. The swimmer of length *L* is placed in a circular area with radius $R=5L$, as shown in Fig. 6. Its goal is to reach the center *O* from any position within the circular area and arbitrarily given orientation. This goal is reflected by defining a reward as

30 $$\begin{aligned} r=-\frac{r_{tip}}{R}, \end{aligned}$$

where $r_{tip}$ is the distance between the head of the swimmer and the center *O*.

The swimmer propels itself by periodically generating a travelling wave propagating from head to tail, as defined by Eqs. (2) and (3). In order to achieve high maneuverability, the swimmer can change the wave amplitude every half swimming cycle. Each selected set of parameters is considered as an action. In this case, $U=1L/s$ is chosen as the characteristic velocity. The period is fixed at $TU/L=1.0$; the amplitude action base is defined as ${\theta }_{lmax}=0^\circ$, $20^\circ$, $40^\circ$, $60^\circ$, $80^\circ$, $100^\circ$, $120^\circ$, $140^\circ$ and $160^\circ$; and the wavelength is fixed at $\lambda =L$. This parameter set forms an action base of 9 components.

**Figure 7:**
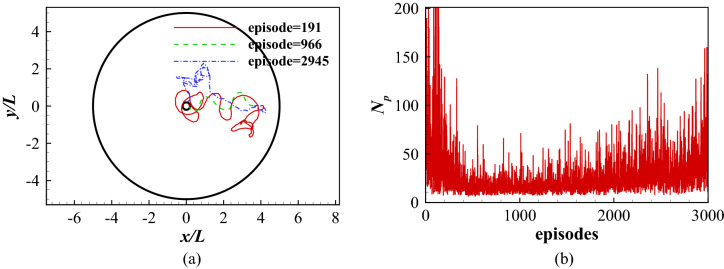
Point-to-point swimming with only the dynamics of the fish considered as the state: (**a**) the traces of the head for at different learning stages; and (**b**) the total number of periods the fish maintains in the swimming area for all episodes considered.

The state is an important component in the DRQN. Theoretically, it should include the information of the swimmer and the ambient flow. The information of the swimmer includes the body waveform, position, pitch angle, velocity, angular velocity, acceleration and angular acceleration of the body. The flow information includes the flow velocity and pressure in the whole flow field. The historical evolution of the flow should also be considered. Therefore, it is impossible to consider the flow information as a simple definition of the state. One way to resolve this problem is to consider the information of the swimmer only as that in the work of Gazzola et al.^21^, Novati et al.^22^ and Verma et al.^23^ . However, ignoring the flow information will make the learned policy inaccurate as shown in Fig. 7a, where only the body waveform, position and pitch angle are considered in all the states. The fish is able to reach its destination in different stages of the learning process, but the path is highly diverse and complicated, and not improving with learning. Figure 7b shows the total number of periods ($N_p$) the fish takes to reach its destination for all learning episodes. In the first 500 episodes, the fish dramatically decreases its time needed to reach the goal, indicating the fish is continuously learning and improving its swimming policy. However, after 500 episodes, the required time grows gradually, indicating the policy is getting worse as the learning progresses. This is because the defined states without considering the flow information is not able to capture the variability of the environment.

Here, we propose a method to consider the influence of the flow information in the states without having to deal with the complexity of the flow. Considering the flow is developed from the historical actions and fish dynamics, it is partially reflected by the dynamics and actions of the swimmer in the past time. If the whole historical dynamics and actions are considered in the states, the flow information is naturally included. However, tracking the whole historical dynamics and actions is memory and time consuming and not necessary since the far history only has minor influence on the flow dynamics at current instant. Our simulations show that only considering the historical dynamics and actions of the fish in the last 4 periods is enough to capture the flow dynamics. In order to further reduce the complexity, accelerations are not considered in the state. The state is thus defined by a tuple

31 $$\begin{aligned} s_n = \left[ \begin{array}{cccccc} (r_{tip})_n, &{} (\theta _{tip})_n, &{} (\bar{u}_{cxl})_n, &{} (\bar{u}_{cyl})_n, &{} \bar{\omega }_n, &{} \\ (r_{tip})_{n-1}, &{} \quad (\theta _{tip})_{n-1}, &{} \quad (\bar{u}_{cxl})_{n-1}, &{} \quad (\bar{u}_{cyl})_{n-1}, &{} \quad \bar{\omega }_{n-1}, &{} \quad a_{n-1} \\ \ldots , &{} \ldots , &{} \ldots , &{} \ldots , &{} \ldots , &{} \ldots , \\ (r_{tip})_{n-8}, &{} \quad (\theta _{tip})_{n-8}, &{} \quad (\bar{u}_{cxl})_{n-8}, &{} \quad (\bar{u}_{cyl})_{n-8}, &{} \quad \bar{\omega }_{n-8}, &{} \quad a_{n-8} \\ \end{array} \right] , \end{aligned}$$

where $\theta _{tip}$ is the orientation angle of the swimmer relative to $r_{tip}$ (as defined in Fig. 6), $\bar{u}_{cxl}$ and $\bar{u}_{cyl}$ are the mean swimming velocities over half a period in the $x_l$ and $y_l$ directions, and $\bar{\omega }$ is the mean angular velocity over half a period. For a real fish, $r_{tip}$ and $\theta _{tip}$ can be directly sensed by the eyes, while $\bar{u}_{cxl}$, $\bar{u}_{cyl}$ and $\bar{\omega }$ can be sensed by the lateral line system^11,70,71^. Therefore, it is reasonable to use these quantities to define the state.

**Figure 8:**
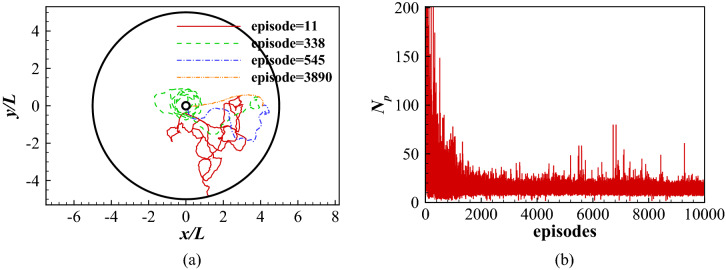
Point-to-point swimming by considering the influence of the flow dynamics in the states: (**a**) the traces of the head during different learning stages; and (**b**) the total number of periods the fish maintains in the swimming area for all episodes considered.

The simulation is performed for a Reynolds number of $Re=\rho UL/ \mu =1000$. It should be noted that this is not a typical Reynolds number for an adult fish. Instead it is for a juvenile fish less than 5cm swimming in this scope. This Reynolds number is used to reduce the computational cost, while such setup is sufficient to demonstrate the effectiveness of the coupled DRQN and IB–LBM. The computational domain of $50L\times 50L$ is divided into 7 blocks with about $41.3\times 10^3$ initial points. The minimum nondimensional grid spacing is $\Delta x/L=\Delta y/L=0.01$ near the inner boundaries and the nondimensional time step size is $\Delta t U/L=0.01$. The simulation requires about 2.52s of CPU time per nondimensional time unit $tU/L=1.0$. The learning parameters are set to $\alpha =0.001$ and $\gamma =0.99$, while $\epsilon$ decays from 1 to 0.05 gradually. These parameters are chosen to ensure the stability of the learning process.

The learning process is divided into a series of episodes. In each episode, the initial position $(r_{tip})_0$ is randomly chosen between *L* and 5*L* and the initial orientation $(\theta _{tip})_0$ randomly varies between $-90^\circ$ and $90^\circ$. The position and orientation of the swimmer are then determined by the FSI with the actions. Once the swimmer exceeds the circular area or reaches the center or reaches 200 periods in the area, the episode ends and another starts. Figure 8 shows the traces of the head during different learning stages and the total number of swimming periods the fish maintains in the swimming area for all episodes considered. As shown in Fig. 8a, the swimmer swims randomly in episode 11. Nevertheless, after a trial and error exploration period, it learns to adjust its orientation and swims around the center *O* (episode 338). After learning for 545 episodes, it successfully finds a tortuous path to reach the center *O*. However, at episode 3890, it has learned how to directly swim towards its destination. This is further demonstrated by Fig. 8b, from which it is found that in the first 2000 learning episodes, the total number of swimming periods decreases rapidly. After around 2000 episodes, the total number of swimming periods remains at a low value, indicating the swimmer has found an efficient way to reach its goal.

**Figure 9:**
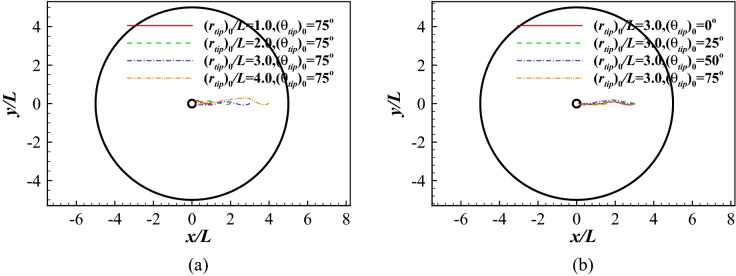
Point-to-point swimming: (**a**) the traces of the head for different initial distance $(r_{tip})_0$; and (**b**) the traces of the head for different initial orientations $({\theta }_{tip})_0$.

Figure 9 presents the traces when the fish swims to its destination with different $(r_{tip})_0$ and $(\theta _{tip})_0$ after learning for 10,000 episodes. 8 cases are studied. In the first 4 cases, $(\theta _{tip})_0$ is fixed at $75^\circ$ while $(r_{tip})_0$ takes on the values 1*L*, 2*L*, 3*L* and 4*L*. In the other 4 cases, $(r_{tip})_0$ is fixed at 3*L* while $(\theta _{tip})_0$ takes on the values $0^\circ$, $25^\circ$, $50^\circ$ and $75^\circ$. In all cases, the fish directly swims to its destination with a very short path.

**Figure 10:**
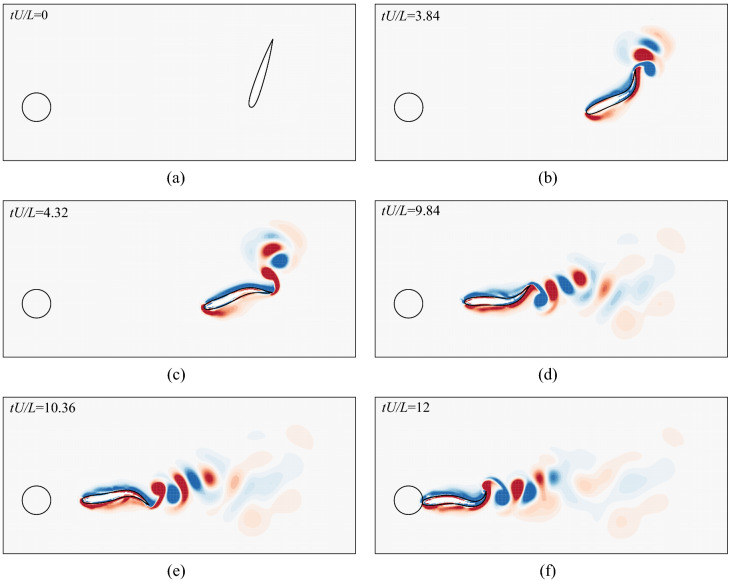
Vorticity contours behind the fish during point-to-point swimming at six typical instants: (**a**) $tU/L=0$, (**b**) $tU/L=3.84$, (**c**) $tU/L=4.32$, (**d**) $tU/L=9.84$, (**e**) $tU/L=10.36$, and (**f**) $tU/L=12$. The range of the vorticity contours is from $-4$ to 4. Note that the flow inside the body-occupied region is introduced by the IB–LBM which however does not affect the solution in the physical region. Flow visualization is achieved by using Tecplot 360 EX 2015 R2 (https://www.tecplot.com).

Figure 10 shows the vorticity contours at different instants while the fish swims to its destination with an initial distance of $(r_{tip})_0=3L$ and an initial orientation of $({\theta }_{tip})_0=75^\circ$. Initially the fish is at rest with the destination to its right (see Fig. 10a). Then it undulates with large right amplitude (see Fig. 10b) and small left amplitude (see Fig. 10c) to perform a fast right turn. After directly facing the destination, it swims with nearly equal left (see Fig. 10e) and right (see Fig. 10d)amplitudes. At around 12 periods, the fish successfully reaches the destination (see Fig. 10f).

### Rheotaxis

**Figure 11:**
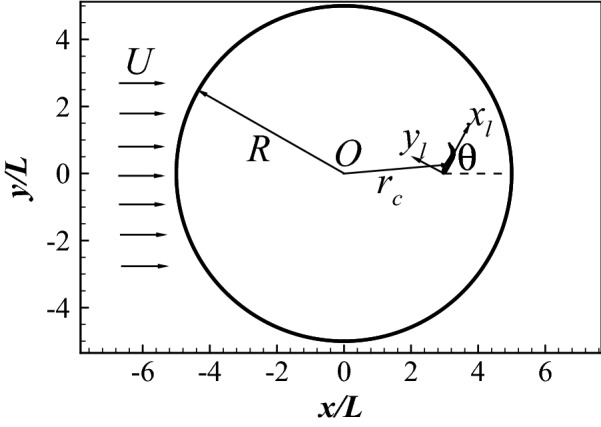
The confined domain of the rheotaxis swimming.

Here we apply the coupled approach to the rheotaxis swimming of a sub-carangiform swimmer in a uniform flow. Its goal is to hold position in a circular area of radius $R=5L$ as shown in Fig. 11 for more than 200 periods. The situation is highly unstable since a small displacement in orientation away from the flow direction could lead to high lateral forces making the agent swim away from its original position. This goal is reflected by defining a reward as

32 $$\begin{aligned} r=-|\bar{\varvec{u}}|, \end{aligned}$$

where $\bar{\varvec{u}}$ is the mean translation velocity of the center of the mass in each half a period.

In this case, the swimmer is able to change both the wave period and amplitude every half swimming cycle. The period action base is defined as $TU/L=0.3$, 0.4 and 0.5; the amplitude action base is defined as ${\theta }_{lmax}=18^\circ$, $35^\circ$ and $55^\circ$, and the wavelength is fixed at $\lambda =L$. This parameter forms an action base of 9 components. The values are chosen carefully so that the fish can perform different maneuvering like acceleration, deceleration and yawing.

Note that the information of the position $r_c$ and orientation $\theta$ is implied in the translational and rotational velocities, and thus is not necessary for the fish to sense. Therefore, the state is simplified to be

33 $$\begin{aligned} s_n = \left[ \begin{array}{cccc} (\bar{u}_{cx})_n, &{} (\bar{u}_{cy})_n, &{} \bar{\omega }_n, &{} \\ (\bar{u}_{cx})_{n-1}, &{} \quad (\bar{u}_{cy})_{n-1}, &{} \quad \bar{\omega }_{n-1}, &{} \quad a_{n-1} \\ \ldots , &{} \ldots , &{} \ldots , &{} \ldots , \\ (\bar{u}_{cx})_{n-8}, &{} \quad (\bar{u}_{cy})_{n-8}, &{} \quad \bar{\omega }_{n-8}, &{} \quad a_{n-8} \\ \end{array} \right] , \end{aligned}$$

where $\bar{u}_{cx}$ and $\bar{u}_{cy}$ are the mean translational velocities of the center of the mass in each half a period parallel and perpendicular to the flow orientation.

**Figure 12:**
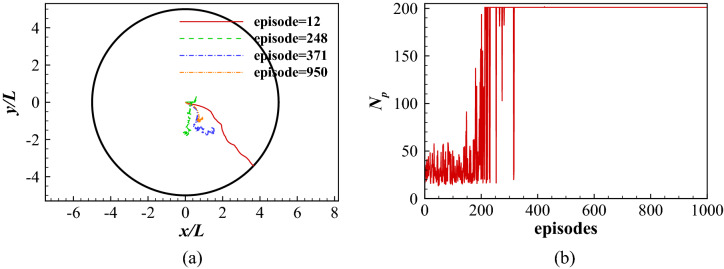
Rheotaxis: (**a**) the traces of the center of the mass during different learning stages, and (**b**) the total number of periods the fish maintains in the swimming area for all episodes considered.

The simulation is performed for a Reynolds number of $Re=\rho UL/ \mu =1000$. The computational domain of $50L\times 50L$ is divided into 7 blocks with about $45.6\times 10^3$ initial points. The minimum nondimensional grid spacing is $\Delta x/L=\Delta y/L=0.01$ near the inner boundaries and the nondimensional time step size is $\Delta t U/L=0.01$. The simulation requires about 2.99s of CPU time per nondimensional time unit $tU/L=1.0$. The learning parameters are set to $\alpha =0.001$ and $\gamma =0.99$, while $\epsilon$ decays from 1 to 0.05 gradually.

The swimmer is initially placed in the center *O* of the swimming area with its initial orientation angle $\theta _{0}$ randomly varying between $-45^\circ \le \theta _{0} \le 45^\circ$. Figure 12 shows the traces of the center of the mass during different learning stages and the total number of swimming periods the fish maintains in the swimming area. As shown in Fig. 12a, the fish is not able to hold position at episode 12. At episode 248 and episode 371, the fish has learned to hold position for more than 200 periods but it still moves around with a very low speed. However, at episode 950, the fish is able to hold position without obvious displacement after a initial adjustment period. In the first approximately 200 episodes (see Fig. 12b), the total number of swimming periods increases rapidly, indicating the fish is learning to hold position. After approximately episode 200, the fish is able to maintain in the swimming area for more than 200 periods, indicating it has found a policy to hold position.

**Figure 13:**
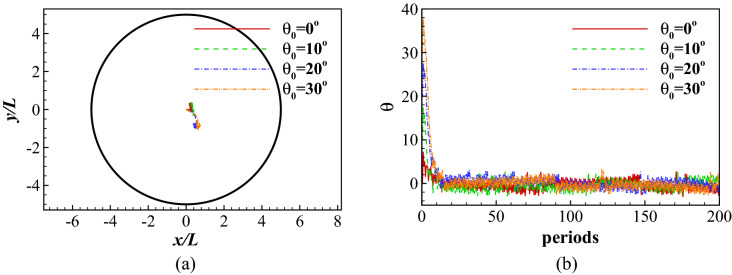
Rheotaxis: (**a**) the traces of the center of the mass for different initial orientations $\theta _{0}$; and (**b**) the time history of the orientation angle for different initial orientations $\theta _{0}$.

Figure 13 compares the traces of the center of the mass and the change of the orientation angle $\theta$ after learning for 1000 episodes. 4 cases are studied with initial oritentation $\theta _{0}$ values of $0^\circ$, $10^\circ$, $20^\circ$ and $30^\circ$. The swimmer holds position for more than 200 periods in all cases as shown in Fig. 13a. As shown in Fig. 13b, it rapidly adjusts its orientation during the first 10 periods to align its body against the flow, and thereafter tries to hold its position.

**Figure 14:**
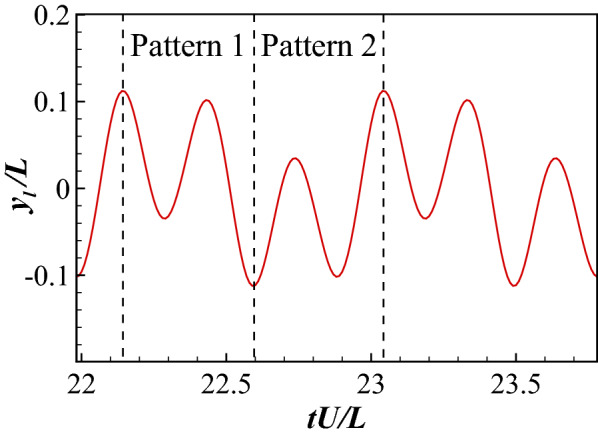
The lateral movement of the tail during rheotaxis.

**Figure 15:**
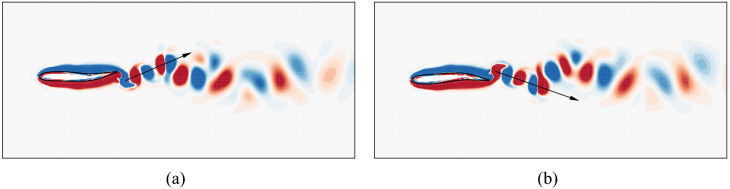
Wake vorticity contours during rheotaxis: (**a**) Pattern 1; and (**b**) Pattern 2. The range of the vorticity contours is from $-4$ to 4. Flow visualization is achieved by using Tecplot 360 EX 2015 R2 (https://www.tecplot.com).

The lateral movement of the tail when the swimmer is holding position is presented in Fig. 14. A repetitive undulating pattern is apparent that lasts for 4 flapping periods. In this pattern, we can identify two types of tail movement: the first is continuously increasing the left amplitude (Pattern 1), the second is continuously increasing the right amplitude (Pattern 2). These flapping patterns trigger two types of wake vortices as shown in Fig. 15. In the first type of wake vortices (Fig. 15a), the vortices form a jet wake deflected slightly to the right side of the swimmer, causing it to move to its left and to rotate clockwise slightly. In the second type of wake vortices (Fig. 15b), a leftward deflected jet is formed causing the swimmer to move to the right and to rotate anticlockwise. These patterns of vortices happen in turn, realizing a dynamical balance in the hydrodynamic forces to hold position in the flow.

### Position holding in a Kármán vortex street

Here we apply the coupled approach to the position holding behavior in a Kármán vortex street. The Kármán vortex street is an example of a drag wake, characterized by a repeating pattern of swirling vortices. It is a complex but mostly predictable flow environment. The abundant vortices make the fluid dynamics in different areas highly diverse and unsteady and there is always a certain amount of unpredictable variation in the vortex behaviors^72^. Furthermore, a fish in the Kármán vortex street selectively explores the flow and swims back and forth when slaloming around the incoming vortices^73^, which makes the encountered flow field more variable and unpredictable.

Liao and Akanyeti^50,72,73^ conducted a series of experiments to observe the kinematics of live rainbow trouts in the Kármán vortex street, in which the fish were placed in the wake behind a D-shaped cylinder. They found that the midline kinematics of the fish could be represented as a superimposition of four midlines generated by four motion components: lateral translation, body bending, body rotation and head motion, whose contributions were respectively $67.8\%$, $19.9\%$, $9.0\%$ and $3.3\%$ in terms of the swept area. The frequencies of the tail beats matched the vortex shedding frequency. The body wavelength was approximately $25\%$ larger than the wake wavelength. In addition, the peak-to-peak tail beat amplitude was nearly the same as the diameter of the cylinder.

A D-shaped cylinder of diameter $D=0.3L$ is chosen in our simulation to produce the Kármán vortex street for comparison with the experiment of Liao^50^. The Strouhal number of the vortex street is $St=fD/U=0.1875$ resulting in a non-dimensional vortex frequency $fL/U=0.625$ and period $TU/L=1.6$. The wavelength of the vortex street is around 1*L*. The fish is trained in a rectangular area of $8L \times 4L$. Its goal is to hold its horizontal position in the vortex street for more than 200 periods. The goal is reflected by defining a reward as

34 $$\begin{aligned} r=-|\bar{u}_{cx}|. \end{aligned}$$

In this case, the period action base is defined as $TU/L=1.2$, 1.4, 1.6, 1.8 and 2.0; the amplitude action base is defined as $\theta _{lmax}=16^\circ$, $34^\circ$, $51^\circ$, $72^\circ$ and $97^\circ$; and the wavelength is fixed at $\lambda =1.5L$. These parameter sets are within the range of the observation of Liao and Akanyeti^50,72,73^.

The hydrodynamic forces exerted on the fish are also included in the state to better capture the dynamic nature of the flow field. In order to reduce the complexity, body rotation and head motion are not considered, which is based on the observation by Akanyeti and Liao^72^ who found that nearly $90\%$ of the body motion of a live rainbow trout will be captured by the present model. Therefore, the state is defined by

35 $$\begin{aligned} s_n = \left[ \begin{array}{ccccccc} (x_c)_n, &{} (y_c)_n, &{} (\bar{u}_{cx})_n, &{} (\bar{u}_{cy})_n, &{} (\bar{F}_D)_n, &{} (\bar{F}_L)_n, &{} \\ (x_c)_{n-1}, &{} \quad (y_c)_{n-1}, &{} \quad (\bar{u}_{cx})_{n-1}, &{} \quad (\bar{u}_{cy})_{n-1}, &{} \quad (\bar{F}_D)_{n-1}, &{} \quad (\bar{F}_L)_{n-1}, &{} \quad a_{n-1} \\ \ldots , &{} \ldots , &{} \ldots , &{} \ldots , &{} \ldots , &{} \ldots , &{} \ldots , \\ (x_c)_{n-8}, &{} \quad (y_c)_{n-8}, &{} \quad (\bar{u}_{cx})_{n-8}, &{} \quad (\bar{u}_{cy})_{n-8}, &{} \quad (\bar{F}_D)_{n-8}, &{} \quad (\bar{F}_L)_{n-8}, &{} \quad a_{n-8} \\ \end{array} \right] , \end{aligned}$$

where $\bar{F}_D$ and $\bar{F}_L$ are respectively the mean longitudinal and lateral force in each half a period. Learning parameters are all set the same as in Sect. Point‑to‑point swimming.

**Figure 16:**
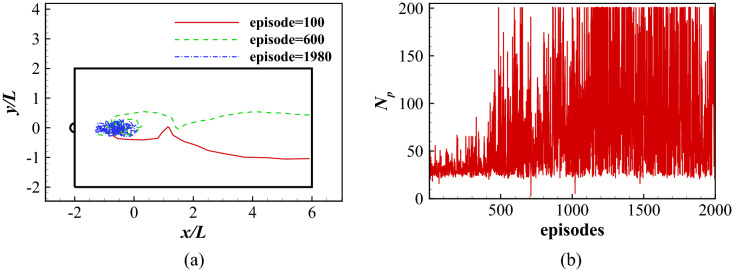
Position holding in a Kármán vortex street: (**a**) the traces of the head during different learning stages; and (**b**) the total number of periods the fish maintains in the swimming area for all episodes considered.

The simulation is performed for a Reynolds number of $Re=\rho UL/ \mu =1000$ or $Re_{cylinder}=\rho UD/ \mu =300$. The computational domain of $50L\times 50L$ is divided into 7 blocks about $52.1\times 10^3$ initial points. The minimum nondimensional grid spacing is $\Delta x/L=\Delta y/L=0.01$ near the inner boundaries and the nondimensional time step size is $\Delta t U/L=0.01$. The simulation requires about 3.11s of CPU time per nondimensional time unit $tU/L=1.0$. The learning parameters are set to $\alpha =0.001$, $\gamma =0.99$, and $\epsilon$ decays from 1 to 0.05 gradually.

**Figure 17:**
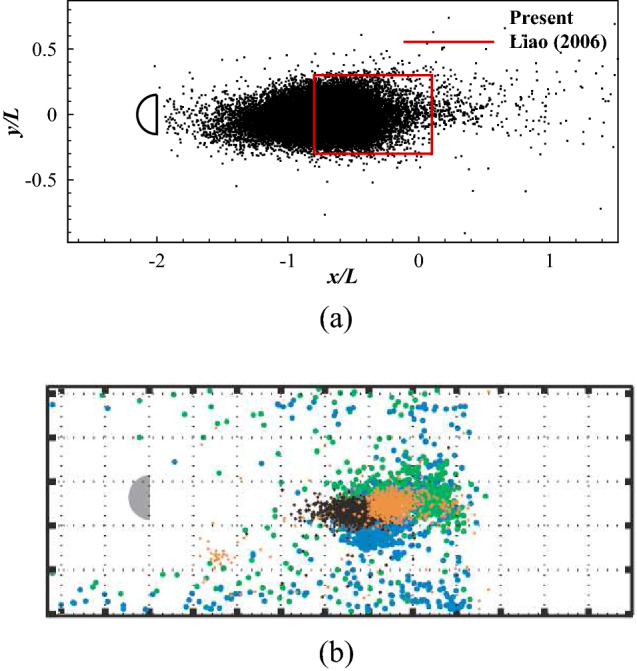
The location of the swimmer swimming in a Kármán vortex street: (**a**) the current result, and (**b**) the experimental observation in Ref.^50^.

The fish is initially placed in the mid-line of the swimming area with its initial distance between the head and the cylinder randomly varying from 1.5*L* to 2.5*L*. Figure 16 shows the traces of the head during different learning stages and the total number of periods the fish maintains in the swimming area for all episodes considered. Figure 16a shows the traces of the head at different learning stages. At episode 100, the fish is not able to hold position and swims out of the area instantly. At episode 600, the fish hold position for several periods but swims out of the area finally. At episode 1980, the fish is able to hold position in a small area for more than 200 periods. As shown in Fig. 16b, in the first approximately 500 episodes, the total number of swimming periods increases rapidly, indicating the fish is learning to hold position. After approximately 500 episodes, the fish is able to hold position in the Kármán vortex street for more than 200 periods, indicating the fish has found an effective swimming policy. Once an efficient swimming policy is achieved, 100 simulations of the swimmer swimming in the wake are conducted. The head location of the swimmer is recorded and shown in Fig. 17a with experimental observation by Liao^50^ in Fig. 17b. It is found that the fish tends to hold position in a small area within the vortex street. Compared with the Kármán gaiting area observed by Liao^50^ in live rainbow trout, the simulation-predicted area where the swimmer tends to stay overlaps the majority of part of that observed in experiment.

The averaged undulation kinematics for 50 successful cases are shown in Table 2 with comparison to the experimental observation of Liao^50^. The Reynolds number of the cylinder in our simulation is 300 compared with 18,000 in the experiment. The resultant tail-beat frequency agrees quite well with that of the experiment but the tail tip amplitude is slightly lower. The undulation wave speed is also slightly slower than that in the experiment due to the smaller wavelength. It should be noted that the typical wake of the D-shaped cylinder in the high Reynolds numbers as observed in experiment is the well-organized turbulence vortex street (see Ref.^17^), which is the foundation of the successful comparison between experiment and simulation as shown in Fig. 17.

**Table 2:** Comparison of undulation kinematics in our simulation and in the observation by Liao^50^.

Variables	Liao (2006)	Present
$Re_{cylinder}$	18,000	300
Tail-beat frequency (*fL*/*U*)	$0.69\pm 0.02$	$0.65\pm 0.01$
Tail tip amplitude ($A_{max}/L$)	$0.19\pm 0.01$	$0.15\pm 0.01$
Body wavelength ($\lambda /L$)	$1.71\pm 0.04$	1.5
Wave speed ($f\lambda /U)$	$1.18\pm 0.01$	$0.98\pm 0.01$

**Figure 18:**
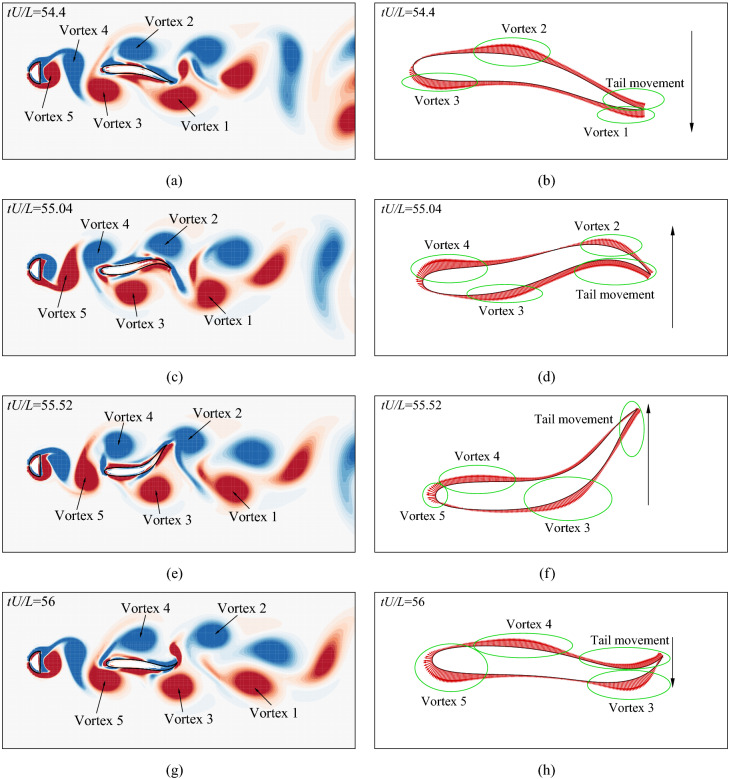
Vorticity contours and pressure distributions on the body surface when the fish is holding position in the Kármán vortex street: (**a**) vorticity at $tU/L=54.4$, (**b**) pressure at $tU/L=54.4$, (**c**) vorticity at $tU/L=55.04$, (**d**) pressure at $tU/L=55.04$, (**e**) vorticity at $tU/L=55.52$, (**f**) pressure at $tU/L=55.52$, (**g**) vorticity at $tU/L=56$, and (**h**) pressure at $tU/L=56$. The range of the vorticity contours is from $-4$ to 4. Flow visualization is achieved by using Tecplot 360 EX 2015 R2 (https://www.tecplot.com).

The vorticity contours and the pressure distributions on the fish surface at different instants when the fish is holding its position in the vortex wake are shown in Fig. 18. It is found that there are at least three vortices that are interacting with the body at any instant. At $tU/L=54.4$ (Fig. 18a and b), vortex 1 is at the left side of the tail and vortex 3 is at the left side of the head, generating high leftwards suction force. Meanwhile, vortex 2 is at the right side of the middle body, generating high rightwards suction force to balance the leftwards suction force at the head and tail. At $tU/L=55.04$ (Fig. 18c and d), the tail is moving from left to right, which leads to a suction force at the left side of the tail, generating leftwards and head-wards thrust. The fish has to balance this leftwards force with its muscles. Meanwhile, vortex 2 has moved to the right side of the tail, inducing a high suction force at the right side which balances the suction force at the left side. In addition, vortex 3 has moved to the left side of the middle body, inducing a backwards drag and a leftwards force which balance the head-wards and rightwards force induced by vortex 4. This facilitates the motion of the tail. At $tU/L=55.52$ (Fig. 18e and f), vortex 3 has moved to the posterior body, inducing a leftwards and a backwards force which balance the head-wards force induced by vortex 5 and the rightwards force induced by vortex 4. At $tU/L=56$ (Fig. 18e and g), the tail is moving from right to left. The leftwards force induced by vortex 3 facilitates this movement. The vortex position is similar to the situation at instants $tU/L=55.04$ while the leftwards force induced by vortex 3 and vortex 5 is balanced by the rightwards force generated by vortex 4 and the tail movement. The backwards force induced by vortex 3 is balanced by the head-wards force induced by vortex 5 and the tail movement. To summarize, the fish can use the vortices to achieve balance and save energy so as to efficiently hold position in the Kármán vortex street.

**Figure 19:**
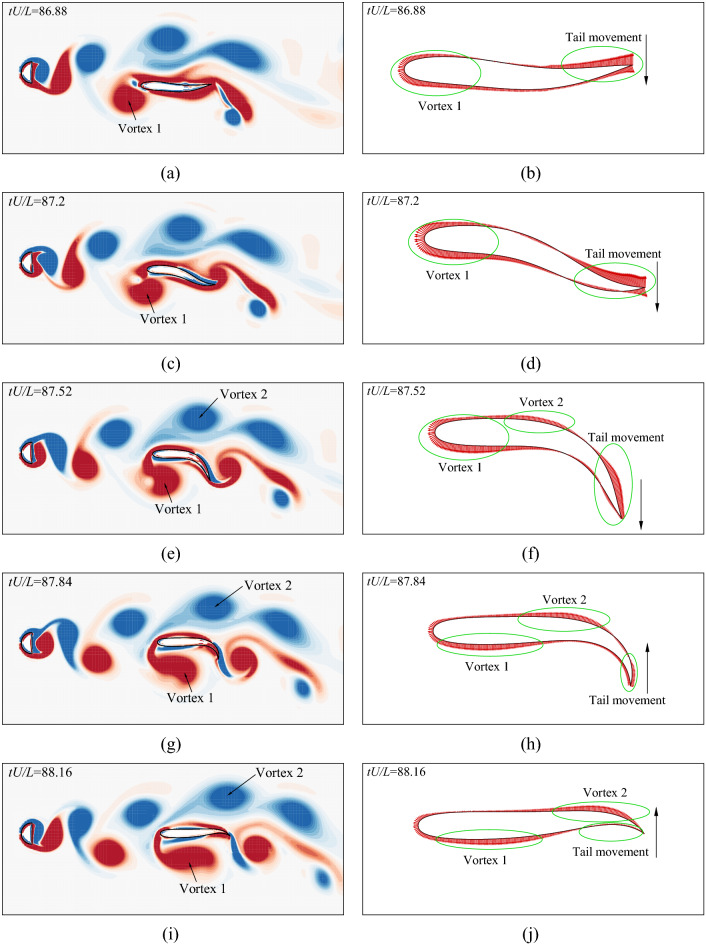
Vorticity contours and pressure distributions on the body surface when the fish is escaping from the vortices: (**a**) vorticity at $tU/L=86.88$, (**b**) pressure at $tU/L=86.88$, (**c**) vorticity at $tU/L=87.2$, (**d**) pressure at $tU/L=87.2$, (**e**) vorticity at $tU/L=87.52$, (**f**) pressure at $tU/L=87.52$, (**g**) vorticity at $tU/L=87.84$, (**h**) pressure at $tU/L=87.84$, (**i**) vorticity at $tU/L=88.16$, and (**j**) pressure at $tU/L=88.16$. The range of the vorticity contours is from $-4$ to 4. Flow visualization is achieved by using Tecplot 360 EX 2015 R2 (https://www.tecplot.com).

However, the fish could occasionally get trapped in the low pressure area of the vortex center. If the swimmer is not able to properly synchronize with the vortices, it would move downstream with the vortex and lose its stability. In this case, the fish must find a way to escape from the vortex in order to hold position for a long period. Figure 19 shows the strategy of how the fish escapes from the vortices. At $tU/L=86.88$ (Fig. 19a and b), 87.2 (Fig. 19c and d) and 87.52 (Fig. 19e and f), the fish is in close proximity to a left side vortex (vortex 1) which induces a high suction force on the left side body. In order to escape from the vortex, the fish performs a fast high-amplitude leftwards flapping to generate high rightwards forces on the tail. At $tU/L=87.84$ (Fig. 19g and h) and 88.15 (Fig. 19i and j), the fish sweeps its tail back to the central area of the vortex street and the leftwards suction force of vortex 1 is partly balanced by the rightwards suction force of vortex 2. Afterwards, the tail is slowly moving from the left side to the right side to avoid generating high leftwards force on the tail.

## Conclusions

The fish adaption behaviors in complex environments have been numerically studied. A recurrent Q-network is first coupled with an immersed boundary–lattice Boltzmann method to simulate the adaption behaviors of a fully self-propelled smart swimmer. Three different behaviors are studied with this swimmer: point-to-point swimming in a quiescent flow, rheotaxis swimming in uniform flow and position holding swimming in a Kármán vortex street. The swimmer utilizes only the position, velocity or acceleration information extracted from the environment to learn to achieve specific tasks. By considering the historical information, the swimmer learns suitable policies to achieve different tasks, demonstrating that deep reinforcement learning is able to extract useful characteristics from flow structures with various complexities. During the point-to-point swimming, the fish performs rapid turning to face the target and then swims directly to it with different initial distances and orientation angles. During rheotaxis swimming, the fish rapidly aligns its body with the uniform flow and holds position for more than 200 periods. Two types of wake vortex patterns are identified for rheotaxis swimming. The vortex patterns produce jet flow in different directions in the wake to facilitate a dynamic balance of the hydrodynamic forces. During position holding in a Kármán vortex street, the fish utilizes the ambient vortices to achieve balance and save energy. The robust position holding in the Kármán vortex street only happens in a specific flow area which is in reasonable agreement with the experimental observation of Liao^50^. Highly asymmetrical corrective undulation is performed when fish is trapped in the vortices, which enables the fish to escape from the vortex center and hold its position or maintain its stability.
